# Enhancing Utilisation of Intraosseous Vascular Access in Cardiac Arrest Management: Insights From a Retrospective Study

**DOI:** 10.7759/cureus.74710

**Published:** 2024-11-28

**Authors:** Hashim S Vakil, Maisha Ahmed

**Affiliations:** 1 Trauma and Orthopaedics, Southampton General Hospital NHS Foundation Trust, Southampton, GBR; 2 General Medicine, Southampton General Hospital NHS Foundation Trust, Southampton, GBR

**Keywords:** cardiac arrest, emergency medicine and trauma, intraosseous access, resus, resuscitation

## Abstract

Intraosseous (IO) vascular access is a crucial intervention during cardiac arrest scenarios, providing a reliable route for emergency medication administration and fluid resuscitation in the absence of a stable intravenous (IV) line or central line. Despite its established efficacy, our retrospective study at Southampton General Hospital revealed under-utilisation rates of IO insertions during cardiac arrests. Among 131 patients studied over a year, only a minority received IO lines. Our findings underscore the need for improved awareness, training, and institutional support to optimise the use of IO vascular access in critical care settings. Our study also highlights, within the limitations of this study, the positive effect an IO has when utilised effectively, in the absence of an IV, in relation to Return Of Spontaneous Circulation (ROSC) rates highlighting and supporting the usage of IO lines in the event an IV is unattainable or difficult to insert.

## Introduction

Intraosseous (IO) vascular access is the ‘placement of a specialised hollow bore needle through the cortex of a bone into the medullary space for infusion of medical therapy and laboratory tests’ [1]. It is most notably utilised during the most acute and emergent situations in a patient’s healthcare including a cardiac arrest scenario especially when the absence of stable venous access would delay lifesaving medications in the acute scenario. First introduced by Dr Cecil K Drinker in 1922 and later re-introduced in the mid-1980s as an alternative route for blood, drug and fluid administration [2], it has been an essential tool and skill often utilised by healthcare professionals in the acute management of highly unwell patients.

Intraosseous vascular (IV) access stands as a pivotal and dependable procedure in the management of cardiac arrest scenarios. This method ensures healthcare professionals with a reliable means of administering emergency medications and conducting necessary tests, thereby facilitating effective patient care during critical moments. It is stated by the Resuscitation Council UK in Advanced Life Support (ALS) that after two failed intravenous access attempts an IO line should be considered [3].

I undertook a study to assess our institution’s utilisation rates of IO insertions during cardiac arrests and to assess if IO lines, in the absence of a patent and stable intravenous line, increase the rates of Return Of Spontaneous Circulation (ROSC).

## Materials and methods

The data were collected retrospectively looking at all the cardiac arrests at Southampton General Hospital. For the study, a total of 186 patients’ cases were studied between April 2022 and April 2023.

The inclusion criteria for this study are as follows: 1) Must be a true cardiac arrest. We were not considering or studying Medical Emergency Team calls for this study; 2) The cardiac Arrest had to have taken place between April 2022 and April 2023; 3) There had to be clear documentation of the resuscitation process scanned onto the patient’s online scanned notes; 4) Paediatric as well as adult patients were included in the audit.

There were a total of 186 patients and out of those 131 patients met the inclusion criteria for the audit.

The data of interest were the length of the cardiac arrests, when the first medication or intravenous bolus of fluid was administered, did the patient survive the arrest, whether was there IV access for the arrest and was an IO line inserted. A decision was made if an IO line may have been considered for the arrest. This was based on the resuscitation council’s advice on IO insertions which is that an IO line should be considered after two intravenous attempts have failed. Although not clearly documented in most arrests, an assumption (which had been discussed with our resuscitation team) was made that if no medication had been inserted within 10 minutes of the arrest that there was no stable access for the medication to go in, hence no intravenous access and therefore IO access should have been considered. Similarly, had an arrest gone on for 15 or more minutes an IO line should have been considered as having more access allows for more medications to be inserted at any given moment.

Following the study of the data mentioned above, a questionnaire was distributed to practitioners, both medical and nursing, questioning their knowledge of the IO lines as well as their confidence in utilising them.

The main question from the study was first, does the usage of an IO line, in the absence of an IV within a suitable time during a cardiac arrest, as detailed above, have a positive outcome or effect on the rate of ROSC? If there was a positive outcome, why are we, as a trust, not utilising the IO lines as much as possible given the more favourable outcome?

## Results

A total of 186 patients who had a crash call put out by their respective medical and surgical teams from April 2022 to April 2023 were studied. Amongst these 131 patients met the inclusion criteria for the study. Of the 131 patients that met the inclusion criteria, 121 were adults and 10 were paediatric patients. The average arrest time for an adult patient included in this study was 23 minutes and for a paediatric patient, it was 33 minutes.

In adult patients, only 6.6% (n=8) of adult patients who suffered a cardiac arrest had an IO line inserted. Of those who did not receive an IO line (n=113), 55.8% (n=63) patients met the requirements to have received an IO line.

Regarding mortality of patients in the study, of the eight patients that received an IO line there was a 62.5% (n=5) ROSC rate. Comparatively, in the 113 adult patients who did not receive an IO line during their arrests, there was a 37.2% (n=42) ROSC rate.

Based on the results seen in adult patients, an odds ratio was calculated for the ROSC rates in adults who had an IO line inserted. This was calculated to be 2.817, indicating a moderate effect of IO lines in relation to ROSC rates.

Of the 10 paediatric patients included in the study, 70% (n=7) had an IO line inserted. For those that had the IO line inserted, in only 28.6% (n=2) was ROSC achieved and in those who did not receive the IO line 66.7% (n=2) ROSC was achieved.

**Figure 1:**
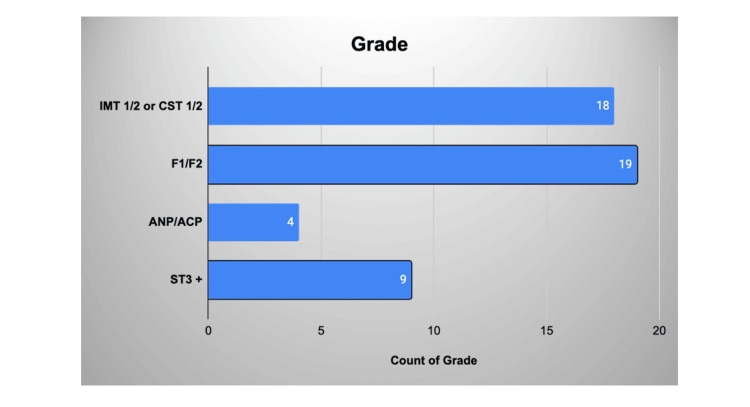
Distribution of grades of practitioners filling out the questionnaire IMT: Internal Medical Trainee; CST: Core Surgical Trainee; ANP: Advanced Nurse Practitioner; ACP: Advanced Care Practitioner; ST3: Specialist Trainee (Registrar)

Fifty practitioners had completed the questionnaire asking them questions regarding their confidence in utilising the IO vascular access (Figure 1).

**Figure 2:**
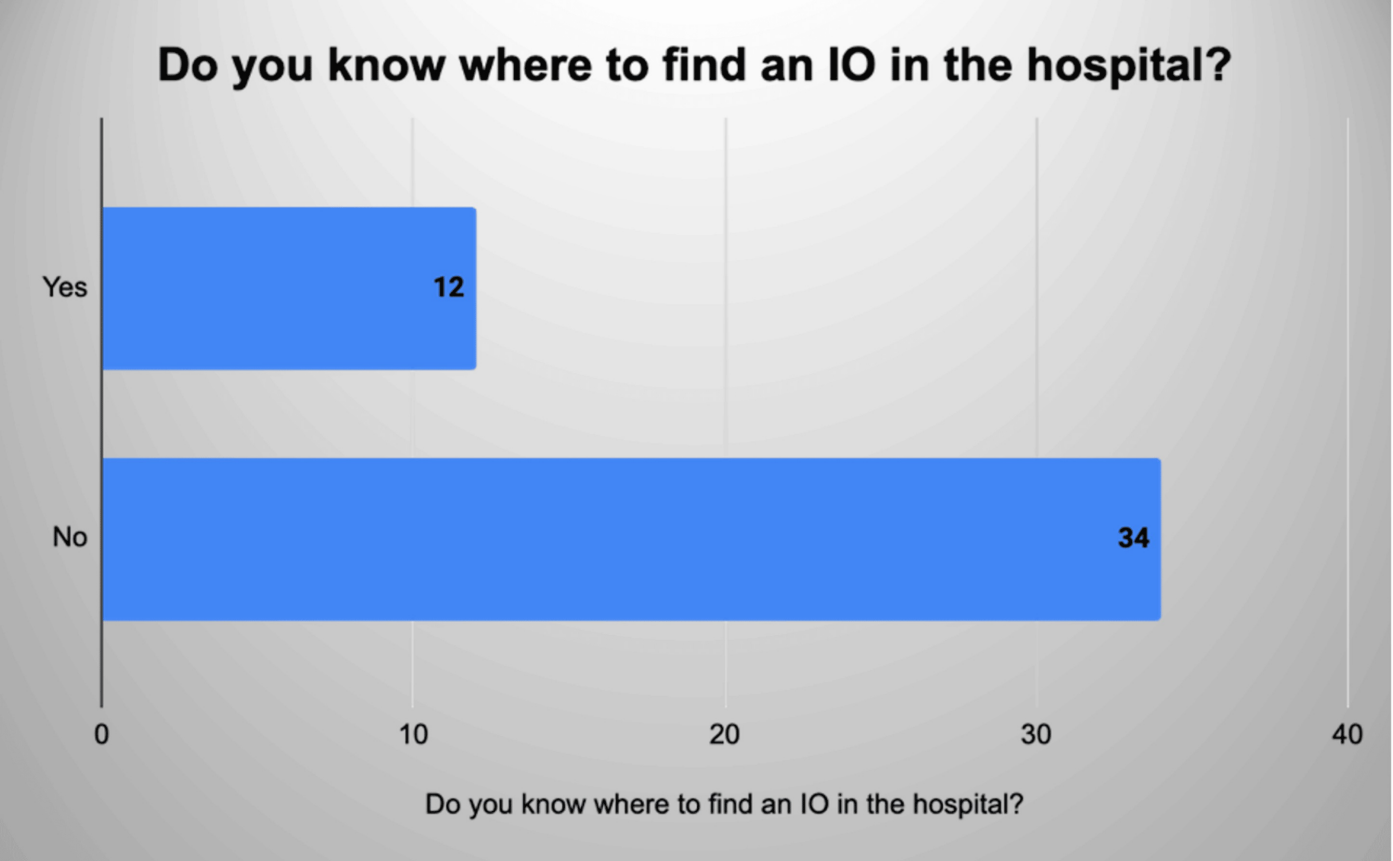
Distribution of grades of practitioner’s response to the question: Do you know where to find an IO in the hospital? IO: intraosseous

Seventy-six per cent of them had never inserted an IO in an adult and only 10% of those that filled in the questionnaire had placed an IO for a paediatric patient. Seventy-eight per cent felt as though they needed more practice with an IO and 73.9% were unsure where to find an EZIO in the hospital (Figure 2).

**Figure 3:**
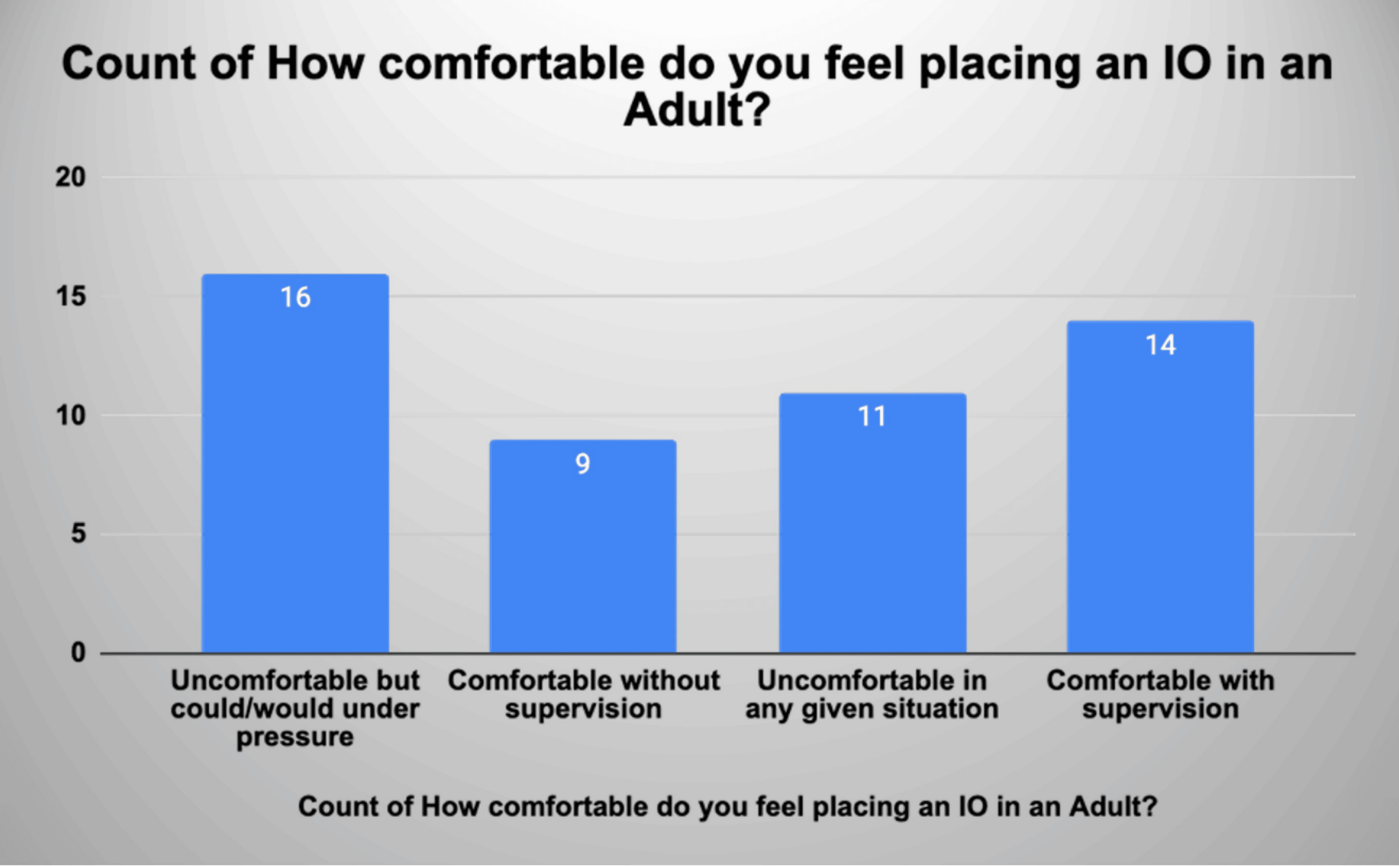
Distribution of grades of practitioner’s response to the question: How comfortable do you feel placing an IO in an adult? IO: intraosseous

Fifty-three per cent of practitioners who completed the form felt uncomfortable putting an IO in, however, 59% of those who felt uncomfortable felt they could do it under supervision. Only 18% of those who filled in the questionnaire felt comfortable inserting an IO line without any supervision (Figure 3).

## Discussion

In this study, data was collected retrospectively and audited based on documentation in notes. Many factors not included in this study may have a significant influence on a clinician’s ability to access an EZIO. Examples which may impact this ability include where the patient was at the time of arrest. It is known that in Emergency Departments, there are a couple of IO lines available in Resus. However, if the patient was to have an arrest in the radiology department, an EZIO may not be readily available to the team. Having spoken to our Resus team at Southampton General Hospital, every Intensive Care Nurse Practitioner tends to have an EZIO on their person when attending a crash call as they may be found in their cardiac arrest backpacks, which they take with them to every arrest they attend. This should in theory negate the concern that a physician wouldn’t have access to an IO if required and there should be one at an arrest call in a timely fashion.

Equally, when studying the ROSC rates for both non-IO and IO study groups, the study does not consider the comorbidities of the patients involved in cardiac arrest. Many patients who unfortunately suffer a cardiac arrest may have certain comorbidities and diseases that may hinder their chances of ROSC [4]. There are many of these variables that may significantly impact the rate of ROSC, however, it would be almost impossible to stratify or quantify the individual rates of each of these comorbidities and how each one individually affected the chance of ROSC for each patient in this study. Rather than focusing on all factors that may affect ROSC rates, I have decided to focus on the time in which we were able to establish vascular access to provide lifesaving emergency medication.

I note certain publications which document that IO medication delivery is associated with inferior rates of ROSC and longer times to ROSC compared to peripheral intravenous vein insertions [5]. Despite said statements, other studies [6] and the study I present show a potential increase of ROSC when utilising the IO versus when not utilising the IO in the absence of a stable IV or central line. Considerations should be made to the size of the study group, the nature of cardiac arrests and the potential multiple comorbidities associated with patients in the studies. Further large-scale studies should be done to assess the rate of ROSC in patients with early and successful IO vascular lines to determine if IO has a definitive positive outcome in cardiac arrests or not.

Rates of complications following IO insertions have been investigated. It is often taught to clinicians that complications of inserting an IO include, but are not limited to, fracture of the long bones, infection of the skin, infection of the bone and compartment syndrome. Systematic reviews have been published where the complications were minimal with only 0.3% of patients involved in the review having serious complications indicating an excellent safety profile [7]. What was noted in a few papers talking about the IO highlighted the success in utilising the IO lines in certain complicated patients [8] as well as the generic contraindications and potential complications [9].

## Conclusions

This retrospective study indicates that there is statistical evidence, despite the small sample study, that the usage of an IO line in the absence of a stable intravenous line provides a positive impact on the rates of Spontaneous Return Of Circulation following a cardiac arrest. Moreover, the proposition of a multi-centre study emerges as a plausible avenue to juxtapose the utilisation of IO vascular lines in cardiac arrest scenarios and to whether an IO in the absence of an IV truly does increase the rates of ROSC.
